# Supplementary material for: Identification of photoperiod-induced specific miRNAs in the adrenal glands of Sunite sheep (Ovis aries)
Source: Front Vet Sci. 2022 Jul 22;9:888207. doi: 10.3389/fvets.2022.888207 (PMC9354845; doi:10.3389/fvets.2022.888207)
Supplement: Supplementary file 1 [file Data_Sheet_1.ZIP › Supplementary file/miRDeep2 file.zip!/miRDeep2 file/Know_Structure/LP42A2/oar-mir-369.pdf]

miRBase precursor : oar-mir-369
Total read count : 7529
oar-miR-369-5p read count : 966
oar-miR-369-3p read count : 6562
remaining reads : 1

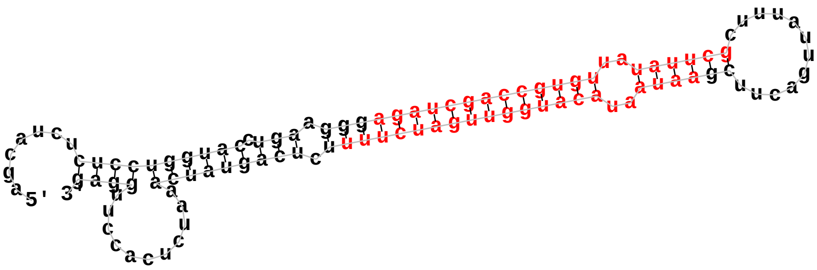

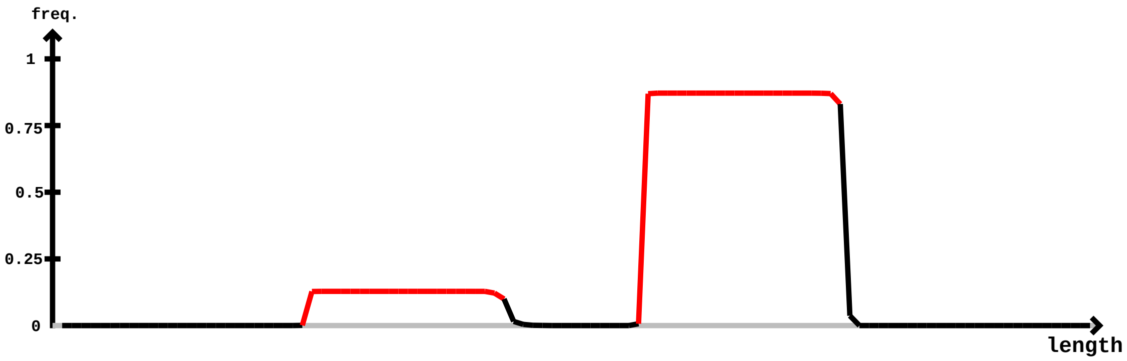


```
                                                   oar-miR-369-5p
                                                                                    oar-miR-369-3p
5'- agcaucucuccugguaccugaagggagaucgaccguguuauauucgcuuuauugacuucgaauaauacaugguugaucuuuucucaguaucaaaucucaccuuggag   -3'    exp
    .......(((((((((.(((.((((((((((((((((..((((((...........))))))..)))))))))))))))).)))))))))..........))))    reads     mm        sample
    .....cucuccugguaccugaaggg..................................................................................    1         0         seq
    ........................gagaucgaccguguuauauucg.............................................................    5         0         seq
    .........................agaucgaccguguuaua.................................................................    1         0         seq
    .........................agaucgaccguguuauauu...............................................................    48        0         seq
    .........................agaucgaccguguuauauuc..............................................................    163       0         seq
    .........................agaucgaccguguuauauucg.............................................................    628       0         seq
    .........................agaucgaccguguuauauucgc............................................................    84        0         seq
    .........................agaucgaccguguuauauucgcu...........................................................    25        0         seq
    .........................agaucgaccguguuauauucgcuu..........................................................    4         0         seq
    .........................agaucgaccguguuauauucgcuuu.........................................................    4         0         seq
    ..........................gaucgaccguguuauauuc..............................................................    3         0         seq
    ..........................gaucgaccguguuauauucg.............................................................    1         0         seq
    ...........................................................gaauaauacaugguugaucuu...........................    5         0         seq
    ...........................................................gaauaauacaugguugaucuuu..........................    47        0         seq
    ............................................................aauaauacaugguugauc.............................    3         0         seq
    ............................................................aauaauacaugguugaucu............................    10        0         seq
    ............................................................aauaauacaugguugaucuu...........................    289       0         seq
    ............................................................aauaauacaugguugaucuuu..........................    5918      0         seq
    ............................................................aauaauacaugguugaucuuuu.........................    274       0         seq
    .............................................................auaauacaugguugaucuu...........................    1         0         seq
    .............................................................auaauacaugguugaucuuu..........................    10        0         seq
    .............................................................auaauaUaugguugaucuuu..........................    1         1         seq
    .............................................................auaauacaugguugaucuuuu.........................    4         0         seq
```
